# Fetal diastematomyelia: MR imaging: A case report

**DOI:** 10.4103/0971-3026.45351

**Published:** 2009-02

**Authors:** Makarand Kulkarni, Mitosh Ruparel, Rajeev Redkar

**Affiliations:** Lilavati Hospital and Research Centre, Mumbai, India

**Keywords:** Diastematomyelia

## Abstract

MRI is increasingly being used in the diagnosis of fetal anomalies suspected on USG. The USG evaluation of fetal spinal anomalies is limited by acoustic shadowing, fetal position and the amount of liquor. Fetal MRI is able to show spinal anomalies well, as in our case of fetal diastematomyelia with a dorsal dermal sinus, suspected on USG at 28 weeks gestation.

## Introduction

Diastematomyelia, also known as split cord malformation (SCM) is a congenital spinal anomaly in which there is longitudinal splitting of the spinal cord. The two hemicords may be separated by a fibrous, bony, or cartilaginous septum.[1] Antenatal detection of cord abnormalities by USG can be limited by acoustic shadowing from the spine, fetal position and the amount of liquor, though it is usually quite accurate in the diagnosis of spina bifida, in experienced hands, when correlated with maternal alpha-fetoprotein levels.[2] MRI is a safe and important tool for confirming the presence of spinal cord abnormalities in a fetus when a suspected spinal deformity has been detected on USG.[1,3–6] Currently, MRI can be performed without maternal or fetal sedation. We report a case of SCM with a dorsal dermal sinus in a 28-week fetus; MRI clearly demonstrated the lesion after routine USG had revealed a suspicious spinal abnormality.

## Case Report

**Figure 1 (A-C):**
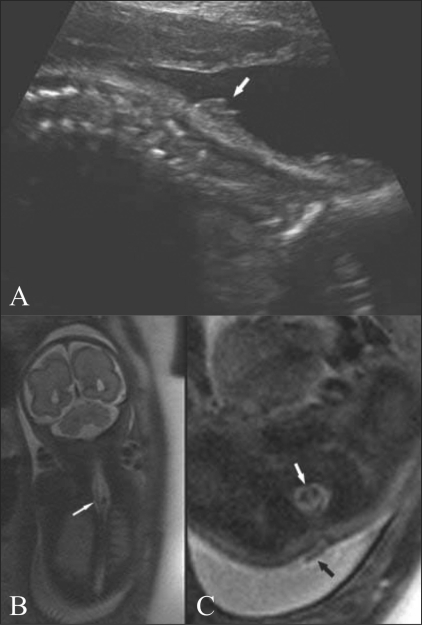
USG (A) of the fetal spine at the cervicodorsal level shows an abnormal soft tissue lesion over the skin (white arrow). Coronal (B) and axial (C), T2W HASTE MRI images show splitting of the cord (arrow in B), and a thick band (white arrow in C) separating the two cords. A band is seen over the skin surface (black arrow in C), with a track seen extending up to the spinal canal

Routine USG in a 28-weeks pregnant woman revealed a spinal abnormality of the fetus. There was widening of the spinal canal in the lower cervical and upper dorsal regions. It was difficult to see the spinal cord due to acoustic shadowing. A superficial soft tissue lesion was seen at the level of the abnormality [Figure 1A]. MRI was performed the next day on a 1.5-Tesla scanner (Symphony, Siemens, Erlangen, Germany). Imaging was performed with a circularly polarized body array coil with the patient in the supine position, using a half-Fourier acquisition single-shot turbo spin echo (HASTE) sequence in the coronal, sagittal, and axial planes of the fetal spine. There was splitting of the spinal cord at the same level as the spinal widening [Figures 1B and C]. The two hemicords were separated by a thick septum [Figure 1C]. A band was seen extending out from the spinal canal at the same level up to the skin surface; this looked like a typical dorsal dermal sinus [Figure 1C]. The distal cord and conus could not be well evaluated as they were very thin. There was no brain anomaly. As 28 weeks' gestation had been completed, the pregnancy could not be terminated.

**Figure 2 (A-C):**
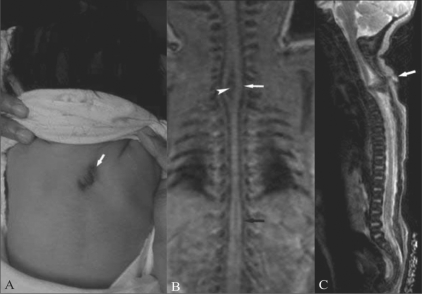
Gross examination of the neonate (A) shows swelling and a tuft of hair (white arrow) in the midline. T1W coronal MRI image (B) of the neonatal spine shows splitting of the spinal cord in the cervicodorsal (white arrow) and lower dorsal (black arrow) regions, with a thick band between the two hemicords (arrowhead). Sagittal T2W HASTE image (C) shows a track (white arrow) extending from the skin surface to the spinal canal.

At birth, examination of the newborn showed a small midline swelling in the upper dorsal region, with a tuft of hair on the skin surface [Figure 2A]. The swelling was soft. There was no discharge. MRI was done on day 2 of the neonatal period. There was diastematomyelia from the C6 to the D2 levels [Figure 2B], with a thick band separating the two hemicords. This band was seen extending up to the pedicle. There was a sinus track extending from the skin surface up to the spinal canal. [Figure 2C]. There was no intraspinal lipoma. The cord was reunited below the D2 level and then was split again from the D8 to the D11 levels [Figure 2B]; there was no septum between the two hemicords at this level. The two hemicords were asymmetrical. There was no cord tethering. The conus ended at the L4 level.

## Discussion

The use of MRI in obstetrics began in 1983,[7] initially being limited to the assessment of maternal and placental abnormalities. In 1990, after the development of single-shot rapid acquisition sequences with refocused echoes, the use of MRI in fetal imaging increased. These T2W sequences, like HASTE, have a very short acquisition time, effectively eliminating the effects of fetal movements.[7] Because of high contrast resolution and the ability to detect sonographically occult structures such as the spinal cord, fetal MRI is an important tool in those cases in which a fetal anomaly is suspected on prenatal USG.[1,3–5] Cornelia *et al*, were able to identify additional spinal cord anomalies, such as diastomatomyelia and segmental spinal dysgenesis, in 10% of cases that were referred after some bony spinal abnormality was detected on USG.[1]

In SCM, there is vertical splitting of the spinal cord. Pang *et al.* have proposed a classification for SCM.[8] According to them, type I SCM consists of two hemicords, each contained in a separate dural tube and separated by an osseocartilaginous septum. Type II SCM consists of a single dural sac containing both hemicords; the two hemicords being separated by a nonrigid fibrous septum. Imaging studies rarely show this fibrous septum. This differentiation has surgical importance as type I split cords are technically more difficult to correct and are associated with more surgical morbidity than type II, especially if there is an oblique septum dividing the cord asymmetrically.[9]

Diastematomyelia may be an isolated finding or may be associated with other spinal dysraphisms such as myelomeningocele, meningocele, lipoma, neurenteric cyst, and dermal sinus. The vertebral anomalies associated with diastematomyelia include hemivertebra, with kyphosis or scoliosis. There may be associated renal, rectal, and uterine malformations.[10] The most common location of diastematomyelia is in the thoracolumbar region. Rarely, it can affect the cervicodorsal region. There is even a case report in the literature of a basicranial diastematomyelia.[11]

In our patient, the diastematomyelia was seen at both the cervicodorsal and the dorsolumbar regions. It was type I at the cervicodorsal region and type II in the dorsolumbar region. However, we could not prenatally detect the splitting of the cord in the dorsolumbar region as the spinal cord was thin. There was an associated dermal sinus in this patient.

Prenatal knowledge of spinal cord anomalies is important for prenatal counseling as well as surgical treatment. Since MRI shows these lesions better and with less inter-observer variation than USG, in patients with suspected spinal anomalies, either diagnosed on USG or based on clinical and laboratory criteria, fetal MRI should be used prior to further management.[1]
